# Percutaneous Aspiration and Sclerotherapy as Primary Management for a Symptomatic Benign Adrenal Cyst

**DOI:** 10.1210/jcemcr/luae110

**Published:** 2024-07-10

**Authors:** John R Strikwerda, Scott M Thompson, Travis J McKenzie, Meera Shah, Irina Bancos, Trenton R Foster

**Affiliations:** Department of Radiology, Mayo Clinic College of Medicine and Science, Rochester, MN 55905, USA; Department of Surgery, Mayo Clinic College of Medicine and Science, Rochester, MN 55905, USA; Department of Radiology, Mayo Clinic College of Medicine and Science, Rochester, MN 55905, USA; Division of Endocrine and Metabolic Surgery, Mayo Clinic College of Medicine and Science, Rochester, MN 55905, USA; Division of Endocrinology, Diabetes, Metabolism, & Nutrition, Mayo Clinic College of Medicine and Science, Rochester, MN 55905, USA; Division of Endocrinology, Diabetes, Metabolism, & Nutrition, Mayo Clinic College of Medicine and Science, Rochester, MN 55905, USA; Division of Endocrine and Metabolic Surgery, Mayo Clinic College of Medicine and Science, Rochester, MN 55905, USA

**Keywords:** adrenal cyst, percutaneous sclerotherapy, incidentaloma

## Abstract

Adrenal cysts are a rare benign adrenal pathology. Although the majority of adrenal cysts are asymptomatic, large cysts may present with debilitating symptoms of mass effect. Surgical adrenalectomy or cyst fenestration has been the primary mode of management for such symptomatic cysts, but these interventions can be associated with excessive morbidity, particularly when considered in the context of benign disease. Here, we present a case of a 34-year-old female with a longstanding, growing, benign left adrenal cyst associated with nonspecific abdominal symptoms. After multidisciplinary discussion, the patient was managed with primary ultrasound/fluoroscopic guided percutaneous sclerotherapy of her adrenal cyst. This technique achieved complete cyst resolution that was durable on 7-month follow-up and was associated with significant improvement of the patient's symptoms. This case illustrates the potential for primary percutaneous sclerotherapy for primary management of benign adrenal cysts.

## Introduction

Adrenal cysts represent 1% to 2% of adrenal masses discovered incidentally on cross sectional imaging (1, 2). They are thus rare compared to other adrenal incidentalomas; nonetheless, the sustained increase in abdominal cross-sectional imaging over recent decades makes them an increasingly encountered pathology. Despite this, definitive clinical guidelines for their management are lacking. This case illustrates the potential of percutaneous sclerotherapy as a safe and effective primary management option for symptomatic benign adrenal cysts.

## Case Presentation

A 34-year-old female presented to the endocrine surgery clinic with chronic generalized abdominal pain, flank and back pain, nausea, and vomiting in the context of a slowly enlarging left adrenal cyst. The cyst was first discovered on computed tomography imaging of the abdomen 11 years previous as an incidental finding measuring 3.4 × 3.7 × 3.9 cm. Notably, in addition to the somatic symptoms detailed previously, the presence of the cyst was causing the patient significant anxiety, and she was thus interested in definitive surgical management.

## Diagnostic Assessment

**Figure 1.**
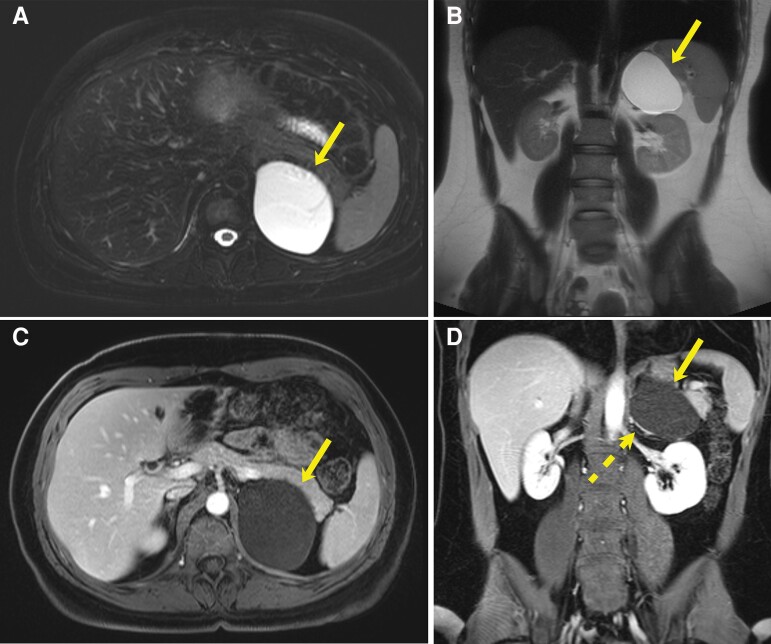
Diagnostic MRI showing a 7.4-cm simple-appearing left adrenal cyst with benign imaging features. (A) Axial T2-weighted fat-saturated, (B) coronal T2-weighted nonfat-saturated, and (C) axial and (D) coronal T1-weighted postgadolinium-enhanced MRI demonstrates a T1-hypointense, T2-hyperintense left suprarenal cyst (solid arrow) without any internal nodularity, septations, or enhancement. (D) The enhancing left adrenal gland along the medial aspect of the cyst demonstrating the “claw sign” with the cyst margin (dashed arrow), thereby confirming the cyst is adrenal in origin.

Magnetic resonance imaging (MRI) of the abdomen completed on presentation revealed that the adrenal cyst had grown and now measured 7.4 × 6.3 × 5.7 cm (Fig. 1). Laboratory evaluation was performed to assess for adrenal hormone excess. This demonstrated a nonfunctioning lesion with an appropriately suppressed cortisol level of <1 mcg/dL (<28 nmol/L) (normal reference range, < 1.8 mcg/dL; < 50 nmol/L) after a 1-mg dexamethasone administration, and 24-hour urine catecholamine and metanephrines that were within normal limits. Aldosterone testing was deferred because the patient was not hypertensive.

## Treatment

Surgical options including adrenalectomy or cyst fenestration were discussed with the patient. However, a less invasive treatment option was desired given the clearly benign pathology on imaging. Additionally, benign adrenal cysts do not typically exhibit significant symptoms, so there was uncertainty whether resection would improve her symptoms. She therefore was referred to interventional radiology for consultation regarding percutaneous aspiration and sclerotherapy, where she was determined to be an appropriate candidate.

**Figure 2.**
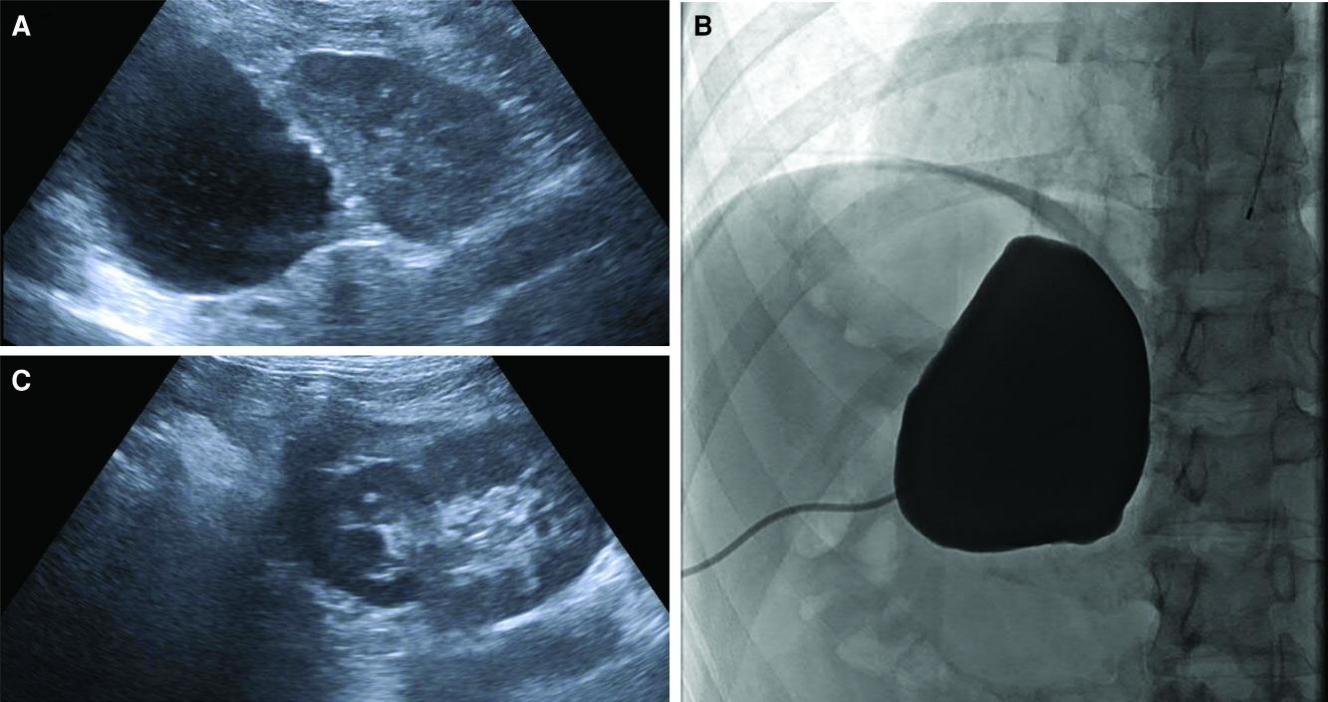
Cyst aspiration and sclerotherapy. (A) Left flank ultrasound demonstrating unilocular anechoic left adrenal cyst. (B) Fluoroscopic images following cyst distention with iodinated contrast. (C) Postaspiration and sclerotherapy ultrasound of left flank demonstrating complete cyst collapse.

The procedure was performed under general anesthesia with endotracheal intubation, and with an arterial line for continuous blood pressure monitoring. The patient was positioned prone to achieve the shortest distance access to the cyst from a retroperitoneal approach. Ultrasound evaluation of the left flank showed a large unilocular hypoechoic suprarenal cyst with debris. Using combined ultrasound/fluoroscopic guidance, a percutaneous 6 Fr locking loop drain was placed in the cyst (Fig. 2) with aspiration of 165 mL of yellow, semiclear fluid with debris. The fluid was sent for cytology, gram stain, and anaerobic and aerobic cultures. Diagnostic sinography was performed in multiple obliquities after maximum distention of the cyst with iodinated contrast to confirm no communication between the cyst and adrenal parenchyma or other structures. Subsequently, 2-agent sclerotherapy was performed with 25 mL of doxycycline mixed with lidocaine and left to dwell for 20 minutes before aspiration; then 25 cc of foamed Sotradecol (3% sodium tetradecyl sulfate mixed 1:4 with room air), left for 20 minutes and aspirated. Following the initial instillation of doxycycline, the patient's blood pressure rose from a baseline systolic blood pressure in the 110s to the 160s mm Hg without tachycardia. The anesthesia team gave labetalol and the patient's blood pressure returned to baseline. Completion ultrasound evaluation showed complete collapse of the left adrenal cyst (Fig. 2). The drain was removed in its entirety. The patient was discharged after 4 hours of postprocedure observation. Follow-up of the cyst fluid was negative for malignancy or infection.

## Outcome and Follow-up

**Figure 3.**
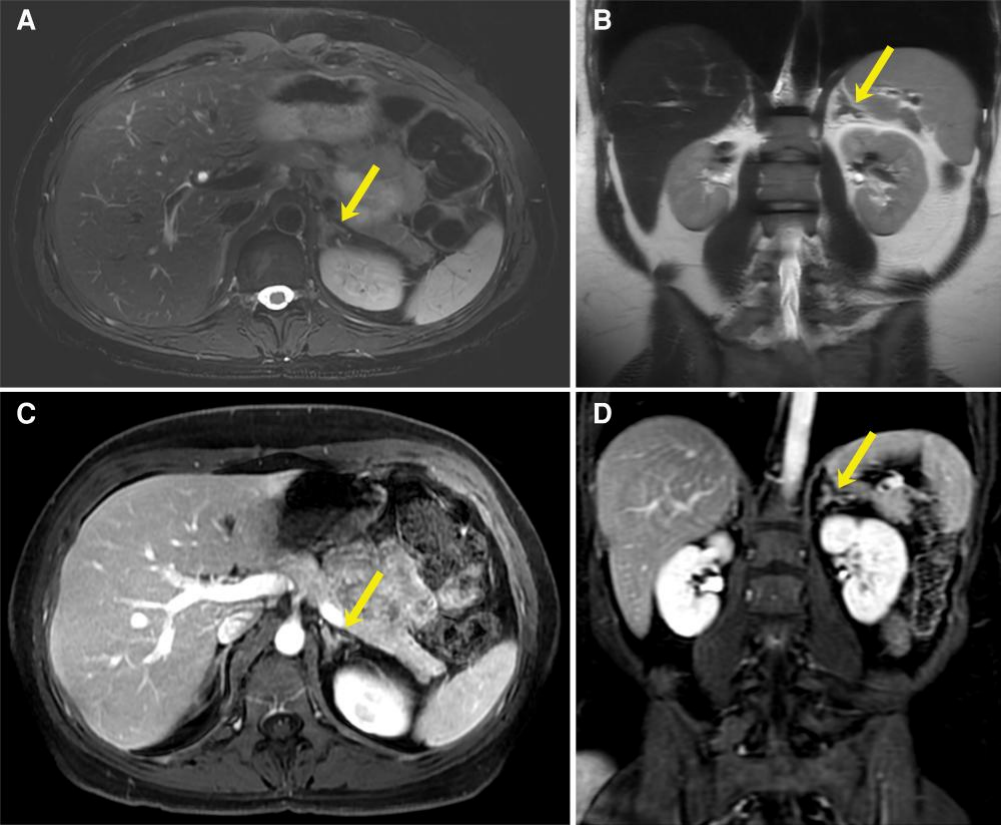
Diagnostic MRI scan showing complete resolution of the left adrenal cyst 7 months following percutaneous aspiration and sclerotherapy. (A) Axial T2-weighted fat-saturated, (B) coronal T2-weighted nonfat-saturated, and (C) axial and (D) coronal T1-weighted postgadolinium-enhanced MRI demonstrates morphologically normal appearing left adrenal gland (arrow) with no residual cyst.

Following sclerotherapy, the patient noted marked symptom improvement, particularly with respect to her left back and flank pain. Symptom resolution lasted 7 months. However, following contraction of COVID-19, she developed recurrent left low back and flank pain. She therefore returned to interventional radiology clinic with these symptoms and anxiety surrounding potential cyst recurrence. This prompted a repeat MRI scan of the abdomen, which demonstrated persistent complete cyst resolution (Fig. 3).

## Discussion

Histopathologically, adrenal cysts are divided into 4 entities: pseudocysts, endothelial cysts, epithelial cysts, and parasitic cysts (3). Of the 4, pseudocysts are the most common and are believed to arise from adrenal hemorrhage that has been subsequently contained by a thick fibrous acellular capsule. Endothelial and epithelial cysts are true cysts with capsules composed of endothelial or lympho-endothelial cells and epithelial cells, respectively. Last, parasitic cysts result from disseminated infection of *Echinococcus granulosus*, often demonstrating characteristic calcified parasitic structures and a cyst capsule containing eosinophilic granulocytes.

Adrenal cysts are predominantly asymptomatic and are therefore most frequently found incidentally on cross-sectional abdominal imaging. In a minority of cases, adrenal cysts can cause symptoms. These are generally symptoms of mass effect such as flank pain, abdominal pain, nausea, and bloating. Importantly, even in the absence of somatic symptoms, incidentally discovered cystic lesions, and their routine surveillance can contribute to significant patient anxiety as demonstrated in our case and elsewhere (4).

Presumably in part because their rarity, adrenal cyst percutaneous aspiration and sclerotherapy have not been widely applied. Conservative management with aspiration of the adrenal cyst alone is more often reported but is plagued by cyst recurrence with or without the return of clinical symptoms (5, 6). In contrast, we identify only 3 reports in the literature where percutaneous sclerotherapy therapy was used: 1 for a large simple adrenal cyst after recurrence following aspiration, 1 as primary management of a parasitic cyst in which partial response was achieved, and another in which percutaneous sclerotherapy was used for cyst recurrence following surgical fenestration (7-9). Thus, surgical management remains the only widely used definitive treatment for benign adrenal cysts. Such a paradigm stands in contrast to management of symptomatic benign cysts originating from other intra-abdominal organs such as liver and kidney where percutaneous sclerotherapy has been widely adopted as an acceptable option for primary management (10-13).

A hesitancy for percutaneous management of adrenal cysts may also be rooted in concern for occult malignancy and occult pheochromocytoma. One early histopathologic study of surgically resected cystic lesions of the adrenal gland demonstrated a rate of neoplasm of nearly 15%, with 5% representing adrenal cortical carcinoma and 5% representing occult pheochromocytoma (14). Such studies appear to support a careful approach to any manipulation of these lesions. However, these surgical cohort studies do not distinguish between a simple cyst and a lesion that is predominantly cystic but with imaging features incongruent with simple cyst. Indeed, the rate of occult malignancy and occult pheochromocytoma quickly approaches zero when cross-sectional imaging demonstrates a simple unilocular, thin-walled cyst without biochemical evidence of hormone excess (15, 16).

Broadly, sclerosing agents can be divided into 3 categories: osmotic agents, detergents, and corrosive agents. A variety of agents within these categories have been used for cyst sclerotherapy including bismuth, povidone-iodine, tetracycline, doxycycline, minocycline, bleomycin, n-butylcyanoacrylate with iodized oil, sodium tetradecyl, hypertonic saline, ethanolamine oleate, and acetic acid (17). Ethanol remains the most commonly used sclerosant for cystic lesion, working primarily as a corrosive agent but also osmotically (12).

In this case, the choice was made to use doxycycline with foamed Sotradecol. This regimen is particularly well tolerated in the authors' practice and has less associated pain and less risk of extracystic penetration compared to an ethanol-based regimen.

When catecholamine release is thought to be likely with adrenal manipulation (ie, mass with demonstrated or suspicion of hormone excess) preoperative α-blockade several days before the procedure is indicated to avoid malignant hypertension. However, in this case, there was little concern for catecholamine excess given a negative laboratory evaluation and characteristic simple cyst features on cross-sectional imaging. Thus, preoperative α blockade was considered but deferred. Nonetheless, a complete anesthesia team was present during the operation and general anesthesia was administered with an arterial line in place for continuous blood pressure monitoring.

In conclusion, we present a case of percutaneous aspiration and sclerotherapy for primary management of a benign nonparasitic adrenal cyst. We achieved complete cyst resolution that is persistent on 7-month follow-up imaging, with associated improvement of the patient's symptoms and anxiety surrounding the cyst's presence and growth. The patient will now be observed clinically, and additional follow up imaging will only be pursued if there are return of symptoms. This case illustrates the feasibility of percutaneous sclerotherapy as a less invasive alternative to conventional surgical therapy for the primary management of benign adrenal cysts.

## Learning Points

- Benign adrenal cysts are a rare yet increasingly encountered pathology without definitive clinical management guidelines.
- Although asymptomatic cysts can be managed with observation alone, cysts presenting with symptoms of mass effect conventionally have been managed surgically.
- This case illustrates the feasibility of percutaneous aspiration and sclerotherapy as a less invasive alternative to conventional surgical therapy for the primary management of symptomatic benign adrenal cysts

## Contributors

All authors made individual contributions to authorship. M.S., T.F., and S.T. were involved in the diagnosis and management of the patient. S.T. performed the percutaneous intervention. J.S. wrote the initial manuscript draft. J.S., S.T., and T.F. prepared manuscript for submission. T.F., S.T., T.M., M.S., and I.B. provided critical review of the manuscript. All authors reviewed and approved the final draft.

## Data Availability

Data sharing is not applicable to this article as no datasets were generated or analyzed during the current study.
